# The global prevalence of *Spirometra* parasites in snakes, frogs, dogs, and cats: A systematic review and meta‐analysis

**DOI:** 10.1002/vms3.932

**Published:** 2022-09-09

**Authors:** Milad Badri, Meysam Olfatifar, Amir KarimiPourSaryazdi, Leila Zaki, Luis Manuel Madeira de Carvalho, Majid Fasihi Harandi, Fatemeh Barikbin, Parisa Madani, Aida Vafae Eslahi

**Affiliations:** ^1^ Medical Microbiology Research Center Qazvin University of Medical Sciences Qazvin Iran; ^2^ Gastroenterology and Hepatology Diseases Research Center Qom University of Medical Sciences Qom Iran; ^3^ Department of Parasitology and Entomology, Faculty of Medical Sciences Tarbiat Modares University Tehran Iran; ^4^ CIISA, Centro Interdisciplinar de Investigação em Sanidade Animal Faculty of Veterinary Medicine, University of Lisbon Lisbon Portugal; ^5^ Research Center for Hydatid Disease in Iran Kerman University of Medical Sciences Kerman Iran; ^6^ Post Graduate Students of Operative Dentistry, Student Research Committee Qazvin University of Medical Sciences Qazvin Iran; ^7^ Metabolic Diseases Research Center, Research Institute For Prevention Of Non‐Communicable Diseases Qazvin University Of Medical Sciences Qazvin Iran

**Keywords:** amphibians, canine, feline, reptiles, *Spirometra*, zoonosis

## Abstract

**Background:**

*Spirometra* infection is aneglected food‐ and waterborne disease with worldwide distribution.

**Objectives:**

The present study aims to estimate the global prevalence of *Spirometra* species in snakes, frogs, dogs and cats.

**Methods:**

Multiple databases (PubMed, Scopus, ProQuest, Web of Science and Google Scholar) were searched for relevant literatures published up to March 2022.

**Results:**

Among 131 data sets (including 113 articles) that met the inclusion, 15 investigations reported *Spirometra* infection in snakes, 23 in frogs, 41 in dogs and 52 in cats. The pooled prevalence (95% confidence interval) in intermediate hosts and definitive hosts was found to be 0.313% and 0.089%, respectively. Based on continent, the infection was most prevalent in Asia for studies on snakes (0.696%) and frogs (0.181%), while Africa (0.224%) and Oceania (0.203%) were the regions with the highest pooled prevalence rates of the infection in dogs and cats, respectively. Among different diagnostic methods, the highest pooled prevalence was related to morphological method for studies on snakes, frog and cats with rate of 0.665%, 0.189% and 0.104%, respectively. Regarding studies on dogs, the highest pooled prevalence was observed for molecular technique (0.101%).

**Conclusions:**

The results presented here revealed the importance of establishing a prevention and control measure focused on protection of aquaculture systems from being contaminated with faeces of dogs and cats, and raising awareness of parasitic zoonotic diseases to decrease the transmission risk.

## Graphical abstract

Spirometra species have been considered as neglected helminths in snakes, frogs, dogs and cats with a more recent growing interest in understanding its prevalence, means for prevention and better treatment, and the phylogenetic relationship of Spirometra in its various hosts. To build on our understanding of the epidemiology of Spirometra. The review and meta‐analysis in the submitted manuscript will contribute to filling this gap in knowledge.

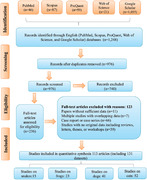

## 1 INTRODUCTION

The worldwide‐distributed pseudophyllidean tapeworms of the genus *Spirometra* inhabit the intestine of canids, felids and other mammals (Kavana et al., 2015, 2014; Le et al., 2017). The life‐cycle involves the crustaceans from *Cyclops* genus as the first intermediate host, amphibians and reptiles, birds and mammals as the second intermediate hosts, carnivores such as dogs and cats as the final hosts and humans as accidental hosts (Kavana et al., 2014). The procercoid larvae develop in *Cyclops* sp., and the plerocercoids develop in amphibians or reptiles (Wiwanitkit, 2005; Yudhana et al., 2019). These plerocercoid larvae (sparganum) are the causative agents of human larval migrans syndromes called sparganosis (spirometrosis), a food and waterborne zoonotic disease, which was firstly described by Manson in 1882 (Anantaphruti et al., 2011; Li et al., 2011; Wang et al., 2014). The disease is endemic in East Asian countries, and has also been reported in populations from Europe, America, Africa and Australia (Wang et al., 2014). So far, there are more than 2000 cases of sparganosis that have been reported in humans worldwide (Kuchta et al., 2015, 2021). However, the number of human cases reported from Eastern and Southeastern Asia is outstanding (Zhang et al., 2020). Humans acquire the infection through drinking water containing procercoid larvae in copepods, consuming raw or undercooked flesh of frogs, snakes, birds and mammals (e.g. pigs) as well as using flesh of frogs or snakes with plerocercoids as poultices on the eyes or open wounds (Li et al., 2011; Q. Liu et al., 2015). The research on sparganosis concentrated more in Asia, where the raw flesh of snake or frog is consumed as a remedy due to the traditional misbelieve and the infection is a serious hazard for humans (MARTA Kołodziej‐Sobocińska et al., 2018; Kuchta et al., 2015). Plerocercoid larvae mostly affect the subcutaneous connective tissues, causing nodules. However, occasionally they invade muscles, the abdominal cavity, eyes, central nervous system, liver, lungs, heart and urinary system (Cui et al., 2011; Kim et al., 2020; Kuchta et al., 2015; Nithiuthai et al., 2004). The migration and proliferation of larvae may result in paralysis, and even death following serious pathologic damages (Oda et al., 2016). There are more than 64 nominal species of *Spirometra* tapeworms (Kuchta et al., 2021). However, only four of them including *S. erinaceieuropaei*, *S*. *mansonoides*, *S. pretoriensis* and *S. theileri* are recognised as valid species (Kuchta et al., 2021; Yamasaki et al., 2021). Epidemiological data are critical for successful application of preventive and control programs against *Spirometra* infection in animals and raises the awareness of the public health hazard caused by these helminthic parasites. In this regard, the present review and meta‐analysis was designed to estimate the pooled prevalence of *Spirometra* tapeworms in snakes, frogs, dogs, and cats in different geographic locations of the world through evaluating available scientific reports.

## 2 MATERIALS AND METHODS

### 2.1 Search strategy

This systematic review and meta‐analysis followed the Preferred Reporting Items for Systematic reviews and Meta‐Analyses (PRISMA) guidelines (Page et al., 2021). Relevant published articles on the prevalence of *Spirometra* in snakes, frogs, dogs, and cats were searched in the following electronic bibliographic databases: PubMed, Scopus, ProQuest, Web of Science and Google Scholar. A systematic search was carried out using the keywords described as follows: *Spirometra*, Sparganosis, Foodborne parasites, Foodborne Diseases, Intestinal helminth, worm, snakes, frogs, dogs, cats, prevalence, frequency, global, worldwide using AND and/or OR Boolean operators. The searching process, evaluation of titles and abstracts and review of the full‐texts were conducted by two independent authors. After removing duplicates and irrelevant records, the reference lists of obtained articles were screened for further studies that were not found in the database search.

### 2.2 Inclusion and exclusion criteria

Full‐text literatures were evaluated for eligibility, if they met the inclusion criteria described below: (1) peer‐reviewed original papers, (2) cross‐sectional studies reporting the prevalence of *Spirometra* in snakes, frogs, dogs, and cats, (3) having accessible full‐text and abstract in English, (4) having total sample size and exact number of positive cases and (5) articles published in English language up to March 2022. Review articles, case reports, case series, publications with non‐original data, letters, editorials and papers with unclear/undetermined results, as well as papers written in other languages were excluded. Moreover, those articles that reported *Spirometra* infection in humans and in animals other than dogs, cats, snakes and frogs were excluded from the analyses of the current study. A Microsoft Excel® version 2016 was used to separately collect the following information that was retrieved from each of the included articles: first author's name, publication year, country where the study was conducted, continent, country‐level income, sample size, number of positive samples, climate, average temperature, annual rainfall, humidity, diagnostic methods including morphological detection and molecular techniques and species of *Spirometra* (Table 1, Table 2).

**TABLE 1:** . Main characteristics of the included studies reporting the prevalence of *Spirometra* in cats, dogs, frog and snakes

No	Reference	Publication year	Country	Continent	Total samples	positive samples	Detection method
**Cats**							
1	Read	1948	United States	North America	14	2	Morphological detection
2	Olsen et al.	1976	United States	North America	82	8	Morphological detection
3	Ryan et al.	1976	Australia	Oceania	146	89	Morphological detection
4	Gregory et al.	1976	Australia	Oceania	107	61	Morphological detection
5	Coman et al.	1981	Australia	Oceania	327	107	Morphological detection
6	Fujinami et al.	1983	Japan	Asia	171	55	Morphological detection
7	Poglayen et al.	1985	Italy	Europe	116	1	Morphological detection
8	Oikawa et al.	1991	Japan	Asia	1064	166	Morphological detection
9	Meloni et al.	1993	Australia	Oceania	33	5	Morphological detection
10	Huh et al.	1993	South Korea	Asia	41	17	Morphological detection
11	Milstein et al.	1997	Australia	Oceania	39	3	Morphological detection
12	Hata et al.	2000	Japan	Asia	326	44	Morphological detection
13	Mcglade et al.	2003	Australia	Oceania	418	2	Morphological detection
14	Scholz et al.	2003	Lao PDR	Asia	55	7	Morphological detection
15	Sohn and Chai	2005	South Korea	Asia	438	181	Morphological detection
16	Zibaei et al.	2007	Iran	Asia	114	4	Morphological detection
17	Palmer et al.	2008	Australia	Oceania	1063	29	Morphological detection
18	Yamamoto et al.	2009	Japan	Asia	1079	90	Morphological detection
19	Castro et al.	2009	Uruguay	South America	4	3	Morphological detection
20	Shin et al.	2009	South Korea	Asia	4	4	Morphological detection
21	Lucio‐Forster and Bowman	2011	United States	North America	1322	5	Morphological detection
22	Headley et al.	2012	British West Indies	North America	55	10	Morphological detection
23	Spada et al.	2012	Italy	Europe	139	2	Morphological detection
24	Al‐Obaidi	2012	Iraq	Asia	55	13	Morphological detection
25	Sabshin et al.	2012	United States	North America	100	1	Morphological detection
26	Borkataki et al.	2013	India	Asia	100	8	Morphological detection
27	Ramos et al.	2013	Brazil	South America	146	6	Morphological detection
28	Hoopes et al.	2013	Canada	North America	635	1	Morphological detection
29	Ngui et al.	2014	Malaysia	Asia	105	2	Morphological detection
30	Rojekittikhun et al.	2014	Thailand	Asia	300	12	Morphological detection
31	Zanzani et al.	2014	Italy	Europe	156	2	Morphological detection
32	Tun et al.	2015	Malaysia	Asia	152	14	Morphological detection
33	Fang et al.	2015	China	Asia	39	13	Morphological detection
34	Zanzani et al.	2015	Italy	Europe	103	1	Morphological detection
35	Rojekittikhun et al.	2015	Thailand	Asia	100	7	Morphological detection
36	Hong et al.	2016	China	Asia	116	47	Morphological detection
37	Pumidonming et al.	2016	Thailand	Asia	180	36	Morphological detection
38	Eslahi et al.	2017	Iran	Asia	12	3	Morphological detection
39	Marques et al.	2017	Brazil	South America	339	1	Morphological detection
40	Blasco et al.	2017	Spain	Europe	423	1	Morphological detection
41	Wyrosdick et al.	2017	United States	North America	76	6	Morphological detection
42	Sedionoto and Anamnart	2018	Thailand	Asia	23	3	Morphological detection
43	Salman et al.	2018	Japan	Asia	351	1	Morphological detection
44	Amoei et al.	2018	Iran	Asia	42	1	Morphological detection
45	Andersen et al.	2018	United States	North America	482	16	Morphological detection
46	Loftin et al.	2019	United States	North America	56	5	Morphological detection
47	Hoggard et al.	2019	United States	North America	103	3	Morphological detection
48	Traversa et al.	2019	Italy	Europe	1000	3	Morphological detection
49	Nagamori et al.	2020	United States	North America	2586	2	Morphological detection
50	Jitsamai et al.	2021	Thailand	Asia	509	8	Morphological detection
51	Tong et al.	2021	China	Asia	135	1	Morphological detection
52	Nath et al.	2022	Bangladesh	Asia	50	19	Morphological detection
**Dogs**							
1	Olsen et al.	1976	United States	North America	2	2	Morphological detection
2	Cho et al.	1981	South Korea	Asia	102	2	Morphological detection
3	Dalimi and Mobedi	1992	Iran	Asia	35	2	Morphological detection
4	Meloni et al.	1993	Australia	Oceania	182	4	Morphological detection
5	Saeki et al.	1997	Japan	Asia	916	1	Morphological detection
6	Hee et al.	1998	South Korea	Asia	304	1	Morphological detection
7	Traub et al.	2002	India	Asia	101	28	Morphological detection
8	Asano et al.	2004	Japan	Asia	772	8	Morphological detection
9	Inpankaew et al.	2007	Thailand	Asia	229	7	Morphological detection
10	Palmer et al.	2008	Australia	Oceania	1400	140	Morphological detection
11	Yamamoto et al.	2009	Japan	Asia	906	9	Morphological detection
12	Lin et al.	2010	China	Asia	31	6	Morphological detection
13	Itoh et al.	2011	Japan	Asia	2365	1	Morphological detection
14	Cardoso et al.	2013	Portugal	Europe	301	1	Morphological detection
15	Schar et al.	2014	Cambodia	Asia	94	20	Morphological detection
16	Ngui et al.	2014	Malaysia	Asia	105	8	Morphological detection
17	Rojekittikhun et al.	2014	Thailand	Asia	500	3	Morphological detection
18	Tun et al.	2015	Malaysia	Asia	227	8	Morphological detection
19	Itoh et al.	2015	Japan	Asia	573	2	Morphological detection
20	Kavana et al.	2015	Tanzania	Africa	59	17	Morphological detection
21	Fang et al.	2015	China	Asia	40	4	Morphological detection
22	Bang et al.	2015	Vietnam	Asia	414	28	Morphological detection
23	Inpankaew et al.	2015	Cambodia	Asia	50	9	Morphological detection
24	Binod et al.	2015	India	Asia	223	98	Morphological detection
25	Hong et al.	2016	China	Asia	229	63	Morphological detection
26	Pumidonming et al.	2016	Thailand	Asia	197	30	Morphological detection
27	Harriott	2016	Australia	Oceania	201	72	Morphological detection
28	Eslahi et al.	2017	Iran	Asia	27	2	Morphological detection
29	Sato et al.	2017	Lao PDR	Asia	34	15	Molecular detection
30	Gillespie and Bradbury	2017	Australia	Oceania	300	4	Morphological detection
31	Binod et al.	2018	India	Asia	223	1	Morphological detection
32	Amouei et al.	2018	Iran	Asia	42	1	Morphological detection
33	Beiromvand et al.	2018	Iran	Asia	167	1	Molecular detection
34	Rusdi et al.	2018	Australia	Oceania	141	5	Molecular detection
35	Little et al.	2019	United States	North America	1202	1	Morphological detection
36	Nagamori et al.	2020	United States	North America	7409	7	Morphological detection
37	Stafford et al.	2020	United States	North America	3006	2	Morphological detection
38	Tong et al.	2021	China	Asia	135	1	Morphological detection
39	Mulinge et al.	2021	Kenya	Africa	65	11	Morphological detection
40	Sobotyk et al.	2021	United States	North America	4692	2	Morphological detection
41	Nath et al.	2022	Bangladesh	Asia	100	62	Morphological detection
**Frogs**							
1	Ooi et al.	2000	Taiwan	Asia	176	18	Morphological detection
2	Berger et al.	2009	Australia	Oceania	243	12	Molecular detection
3	Mao et al.	2009	China	Asia	818	131	Morphological detection
4	WeiMin et al.	2009	China	Asia	671	209	Morphological detection
5	Liu et al.	2010	China	Asia	292	59	Molecular detection
6	Lin et al.	2010	China	Asia	446	75	Morphological detection
7	Young et al.	2012	China	Asia	877	218	Morphological detection
8	Deng et al.	2012	China	Asia	1149	306	Morphological detection
9	Zhang et al.	2014	China	Asia	214	65	Molecular detection
10	Nelli et al.	2014	Armenia	Europe	22	4	Morphological detection
11	Ruijia et al.	2015	China	Asia	153	31	Morphological detection
12	Wei et al.	2015	China	Asia	3482	565	Molecular detection
13	Borteiro et al.	2015	Uruguay	South America	139	2	Morphological detection
14	Hong et al.	2016	China	Asia	1949	229	Morphological detection
15	Zhang et al.	2016	China	Asia	276	55	Molecular detection
16	Wang et al.	2018	China	Asia	511	50	Molecular detection
17	Zhang et al.	2020	China	Asia	386	19	Molecular detection
18	Yudhana et al.	2020	Indonesia	Asia	185	17	Molecular detection
19	Chai et al.	2020	Myanmar	Asia	20	15	Morphological detection
20	Fu et al.	2020	China	Asia	1556	201	Morphological detection
21	Zhang et al.	2020	China	Asia	4078	447	Molecular detection
22	Zhang et al.	2020	China	Asia	386	19	Molecular detection
23	Fu et al.	2022	China	Asia	1556	201	Morphological detection
**Snakes**							
1	WeiMin et al.	2009	China	Asia	3	3	Morphological detection
2	Wang et al.	2011	China	Asia	1160	345	Morphological detection
3	Zhang et al.	2014	China	Asia	6	3	Molecular detection
4	Wang et al.	2014	China	Asia	456	251	Morphological detection
5	Sargsyan et al.	2014	Armenia	Europe	22	2	Morphological detection
6	Pranashinta et al.	2017	Indonesia	Asia	60	41	Morphological detection
7	Kondzior et al.	2018	Poland	Europe	59	2	Molecular detection
8	Wang et al.	2018	China	Asia	346	141	Molecular detection
9	Lu et al.	2018	China	Asia	30	26	Molecular detection
10	Xiao et al.	2019	China	Asia	149	55	Molecular detection
11	Yudhana et al.	2019	Indonesia	Asia	378	192	Morphological detection
12	Liu et al.	2020	China	Asia	375	344	Molecular detection
13	Yudhana et al.	2020	Indonesia	Asia	43	43	Morphological detection
14	Yudhana et al.	2020	Indonesia	Asia	37	21	Morphological detection
15	Yudhana et al.	2021	Indonesia	Asia	55	51	Morphological detection

**TABLE 2:** Sub‐group analysis of the prevalence of Spirometra based on included studies diagnostic method, country‐level income level, genus and species, climate, average temperature, annual rainfall, humidity and continent

					Heterogeneity**		
	Number of studies	Sample size	Number infected	Pooled prevalence (%) (95% CI)	*I* ^2^	*τ* ^2^	*p* Value*
**Variable (cat)**							
Diagnostic method							
**Morphological detection**	52	15,631	1131	0.104 (0.061 to 0.155)	98	0.074	<0.001
Income level							
**High income**	36	13,349	987	0.115 (0.055 to 0.193)	98	0.099	<0.001
**Upper‐middle income**	9	1854	89	0.051 (0.017 to 0.104)	92	0.014	<0.001
**Lower‐middle income**	7	428	55	0.134 (0.039 to 0.273)	87	0.030	<0.001
Genus and species							
***Spirometra erinaceieuropaei***	15	5295	866	0.268 (0.123 to 0.445)	98	0.110	<0.001
***Spirometra mansoni***	5	733	75	0.094 (0 to 0.314)	96	0.049	<0.001
***Spirometra mansonoides***	5	886	39	0.051 (0.001 to 0.164)	84	0.020	<0.001
***Spirometra* spp**.	27	8717	151	0.049 (0.020 to 0.089)	93	0.039	<0.001
Climate							
**Humid continental climate**	15	5594	311	0.142 (0.033 to 0.309)	98	0.131	<0.001
**Tropical savanna climate**	11	4153	441	0.113 (0.046 to 0.204)	96	0.032	<0.001
**Semi‐desert climate**	3	168	8	0.065 (0 to 0.433)	63	0.026	0.070
**Tropical marine climate**	1	55	10	0.181 (0.092 to 0.293)	NA	NA	NA
**Subarctic climate**	1	635	1	0.001 (0 to 0.006)	NA	NA	NA
**Tropical monsoon climate**	2	155	15	0.097(0 to 0.513)	0	0	0.350
**Humid subtropical climate**	9	2426	20	0.024 (0 to 0.114)	73	0.064	<0.001
**Oceanic climate**	8	2188	309	0.203 (0.026 to 0.487)	99	0.105	<0.001
**Tropical rainforest climate**	2	257	16	0.050 (0 to 0.930)	86	0.011	0.007
Average temperature							
**>20°C**	14	2169	146	0.091 (0.043 to 0.154)	92	0.025	<0.001
**10–20°C**	37	12,827	984	0.115 (0.057 to 0.190)	98	0.096	<0.001
**<10**	1	635	1	0.001 (0 to 0.006)	NA	NA	NA
Annual rainfall							
**>1500 mm**	3	307	35	0.128 (0 to 0.733)	94	0.066	<0.001
**1001–1500 mm**	26	10,002	696	0.101 (0.045 to 0.174)	98	0.071	<0.001
**401–1000 mm**	19	5099	379	0.105 (0.030 to 0.217)	98	0.099	<0.001
**<400 mm**	4	223	21	0.103 (0 to 0.357)	85	0.031	<0.001
Humidity							
**>75**	4	742	23	0.030 (0 to 0.115)	89	0.010	<0.001
**40–75**	44	14,666	1087	0.113 (0.063 to 0.175)	98	0.083	<0.001
**<40**	4	223	21	0.103 (0 to 0.357)	85	0.031	<0.001
Continent							
**Asia**	25	5561	756	0.154 (0.081 to 0.244)	96	0.075	<0.001
**Europe**	6	1937	10	0.005 (0.001 to 0.011)	0	0	0.44
**North America**	11	5511	59	0.037 (0.009 to 0.081)	92	0.017	<0.001
**Oceania**	7	2133	296	0.203(0.026 to 0.487)	99	0.105	<0.001
**South America**	3	489	10	0.144 (0 to 1.000)	91	0.235	<0.001
**Variable (dog)**							
Diagnostic method							
**Morphological detection**	38	27,759	668	0.070 (0.032 to 0.120)	98	0.070	<0.001
**Molecular detection**	3	342	21	0.101 (0 to 0.854)	95	0.112	<0.001
Income level							
**High income**	20	25,138	328	0.029 (0.002 to 0.087)	98	0.081	<0.001
**Upper‐middle income**	7	1329	60	0.065 (0.018 to 0.138)	92	0.015	<0.001
**Lower‐middle income**	13	1575	278	0.159 (0.061 to 0.291)	97	0.066	<0.001
**Low income**	1	59	17	0.288 (0.180 to 0.409)	NA	NA	NA
Genus and species							
***Spirometra erinaceieuropaei***	13	7997	266	0.045 (0.006 to 0.116)	98	0.048	<0.001
***Spirometra mansoni***	7	1413	105	0.141 (0 to 0.519)	96	0.201	<0.001
***Spirometra* spp**.	21	18,691	318	0.080 (0.029 to 0.154)	98	0.062	<0.001
Climate							
**Humid continental climate**	14	17,710	148	0.070 (0.006 to 0.195)	97	0.111	<0.001
**Tropical savannah climate**	10	6623	134	0.050(0 to 0.169)	97	0.075	<0.001
**Semi‐desert climate**	4	271	6	0.026 (0 to 0.092)	49	0.004	0.11
**Tropical monsoon climate**	5	640	159	0.250 (0.034 to 0.577)	98	0.070	<0.001
**Humid subtropical climate**	1	301	1	0.003 (0 to 0.013)	NA	NA	NA
**Oceanic climate**	5	2224	225	0.078 (0 to 0.273)	97	0.044	<0.001
**Tropical rainforest climate**	2	332	16	0.050 (0 to 0.510)	57	0.002	0.12
Average temperature							
**>20°C**	15	2621	345	0.162 (0.074 to 0.275)	97	0.059	<0.001
**10–20°C**	26	25,480	344	0.034 (0.008 to 0.077)	97	0.061	<0.001
Annual rainfall							
**>1500 mm**	4	846	106	0.157 (0 to 0.650)	98	0.108	<0.001
**1001–1500 mm**	19	23,654	123	0.037 (0.002 to 0.109)	94	0.094	<0.001
**401–1000 mm**	14	3330	454	0.129 (0.055 to 0.227)	97	0.047	<0.001
**<400 mm**	4	271	6	0.026 (0 to 0.092)	49	0.004	0.11
Humidity							
**>75**	3	746	44	0.057 (0.015 to 0.123)	48	0.001	0.14
**40–75**	33	27,019	628	0.078 (0.031 to 0.143)	98	0.088	<0.001
**<40**	5	336	17	0.051 (0.003 to 0.152)	83	0.013	<0.001
Continent							
**Asia**	28	9141	421	0.076 (0.034 to 0.133)	97	0.055	<0.001
**Europe**	1	301	1	0.003 (0 to 0.013)	NA	NA	NA
**North America**	5	16,311	14	0.071 (0 to 0.755)	80	0.377	<0.001
**Oceania**	5	2224	225	0.078 (0 to 0.273)	97	0.044	<0.001
**Africa**	2	124	28	0.224 (0 to 0.971)	60	0.005	0.11
**Variable (frog)**							
Diagnostic method							
**Morphological detection**	13	9532	1640	0.189 (0.105 to 0.290)	96	0.037	<0.001
**Molecular detection**	10	10,053	1308	0.121 (0.070 to 0.183)	95	0.014	<0.001
Income level							
**High income**	20	19,358	2912	0.143 (0.104 to 0.187)	96	0.015	<0.001
**Upper‐middle income**	1	22	4	0.181 (0.052 to 0.365)	NA	NA	NA
**Lower‐middle income**	2	205	32	0.385 (0 to 1.000)	97	0.260	<0.001
Genus and species							
***Spirometra erinaceieuropaei***	14	13,077	1777	0.130 (0.089 to 0.177)	95	0.011	<0.001
***Spirometra mansoni***	6	4793	953	0.202 (0.135 to 0.280)	96	0.006	<0.001
***Spirometra* spp**.	3	1715	218	0.232 (0 to 1.000)	97	0.218	<0.001
Climate							
**Humid continental climate**	18	18,822	2884	0.163 (0.125 to 0.204)	96	0.010	<0.001
**Tropical monsoon climate**	1	20	15	0.750 (0.542 to 0.910)	NA	NA	NA
**Humid subtropical climate**	2	315	20	0.049 (0 to 0.998)	92	0.018	<0.001
**Oceanic climate**	1	243	12	0.049 (0.025 to 0.080)	NA	NA	NA
**Tropical rainforest climate**	1	185	17	0.091 (0.054 to 0.137)	NA	NA	NA
Average temperature							
**>20°C**	3	381	50	0.273 (0 to 1.000)	95	0.165	<0.001
**10–20°C**	20	19,204	2898	0.146 (0.107 to 0.190)	96	0.015	<0.001
Annual rainfall							
**>1500 mm**	2	361	35	0.096 (0.041 to 0.172)	0	<0	0.74
**401–1000 mm**	20	19,202	2909	0.162 (0.105 to 0.229)	96	0.031	<0.001
**<400 mm**	1	22	4	0.181 (0.052 to 0.365)	NA	NA	NA
Humidity							
**40–75**	23	19,585	2948	0.156 (0.107 to 0.213)	96	0.027	<0.001
Continent							
**Asia**	20	19,181	2930	0.181 (0.052 to 0.365)	96	0.025	<0.001
**Europe**	1	22	4	0.172 (0.119 to 0.232)	NA	NA	NA
**Oceania**	1	243	12	0.049 (0.025 to 0.080)	NA	NA	NA
**South America**	1	139	2	0.014 (0.001 to 0.040)	NA	NA	NA
**Variable (snake)**							
Diagnostic method							
**Morphological detection**	9	2214	949	0.665 (0.349 to 0.915)	97	0.165	<0.001
**Molecular detection**	6	965	571	0.513 (0.132 to 0.884)	98	0.155	<0.001
Income level							
**High income**	9	2584	1170	0.550 (0.252 to 0.830)	98	0.150	<0.001
**Upper‐middle income**	1	22	2	0.090 (0.009 to 0.242)	NA	NA	NA
**Lower‐middle income**	5	573	348	0.790 (0.402 to 0.995)	96	0.102	<0.001
Genus and species							
***Spirometra erinaceieuropaei***	8	2147	918	0.424 (0.142 to 0.736)	98	0.141	<0.001
***Spirometra mansoni***	1	3	3	1.000 (0.712 to 1.000)	NA	NA	NA
***Spirometra* spp**.	6	1029	599	0.752 (0.438 to 0.963)	96	0.092	<0.001
Climate							
**Humid continental climate**	9	2547	1170	0.565 (0.284 to 0.824)	98	0.129	<0.001
**Oceanic climate**	1	59	2	0.033 (0.003 to 0.094)	NA	NA	NA
**Tropical rainforest climate**	5	573	348	0.790(0.402 to 0.995)	96	0.102	<0.001
Average temperature							
**>20°C**	5	573	348	0.790 (0.402 to 0.995)	96	0.102	<0.001
**10–20°C**	9	2547	1170	0.565 (0.284 to 0.824)	98	0.129	<0.001
**<10°C**	1	59	2	0.033 (0.003 to 0.094)	NA	NA	NA
Annual rainfall							
**>1500 mm**	5	573	348	0.790 (0.402 to 0.995)	96	0.102	<0.001
**401–1000 mm**	9	2584	1170	0.550 (0.252 to 0.830)	98	0.150	<0.001
**<400 mm**	1	22	2	0.090 (0.009 to 0.242)	NA	NA	NA
Humidity							
**>75**	1	59	2	0.033 (0.003 to 0.094)	NA	NA	NA
**40–75**	14	3120	1518	0.653 (0.444 to 0.835)	98	0.125	<0.001
Continent							
**Asia**	13	3098	1516	0.696 (0.502 to 0.859)	98	0.100	<0.001
**Europe**	2	81	4	0.049 (0 to 0.656)	0	0.002	*0.33*

* Data analysis was conducted using chi‐square tests.

** Heterogeneity between studies was evaluated using Cochrane's Q test and the *I^2^* statistic.

### 2.3 Quality assessment

A Newcastle–Ottawa Scale was implemented for quality assessment of the included studies (Supplementary Table S1) (Modesti et al., 2016). Scoring was based on three following items: selection (maximum of 5 stars), comparability (maximum of 2 stars) and outcome (maximum of 3 stars) (Badri et al., 2022; Eslahi et al., 2021; Eslahi et al., 2022; Mirzadeh et al., 2021)

### 2.4 Data synthesis and statistical analysis

The pooled prevalence of *Spirometra* in snakes, frogs, dogs and cats reported globally was calculated with 95% confidence interval. Meta‐regression analysis was conducted to evaluate the impact of average temperature, and year of publication on prevalence. Egger's test and Begg's test were applied to specify the possible publication bias. Moreover, publication bias was assessed by the Luis Furuya‐Kanamori (LFK) index and Doi plot (Barendregt & Doi, 2016). An LFK index within the range of ±1, ±2 and outside ±2 was inferred as symmetrical, slightly/minor asymmetrical and significantly/major asymmetrical, respectively, where symmetrical index indicates the absence of publication bias. Freeman‐Tukey double arcsine transformation for the random‐effects model (based on the inverse variance approach for measuring weight) was used to compute the pooled prevalence estimates. Cochrane's Q test and inconsistency index (*I* ^2^ statistics) was used to assess the magnitude of heterogeneity among included studies, with *I* ^2^ values of <25%, 25–75% and >75% were taken as low, moderate and high heterogeneity, respectively. A *p*‐value less than 0.05 was set as statistically significant. All statistical analyses were conducted using the meta‐package of R (version 3.6.1, R Foundation for Statistical Computing, Vienna, Austria) (Team, 2020).

## 3 RESULTS

### 3.1 Search results and study selection

**FIGURE 1:**
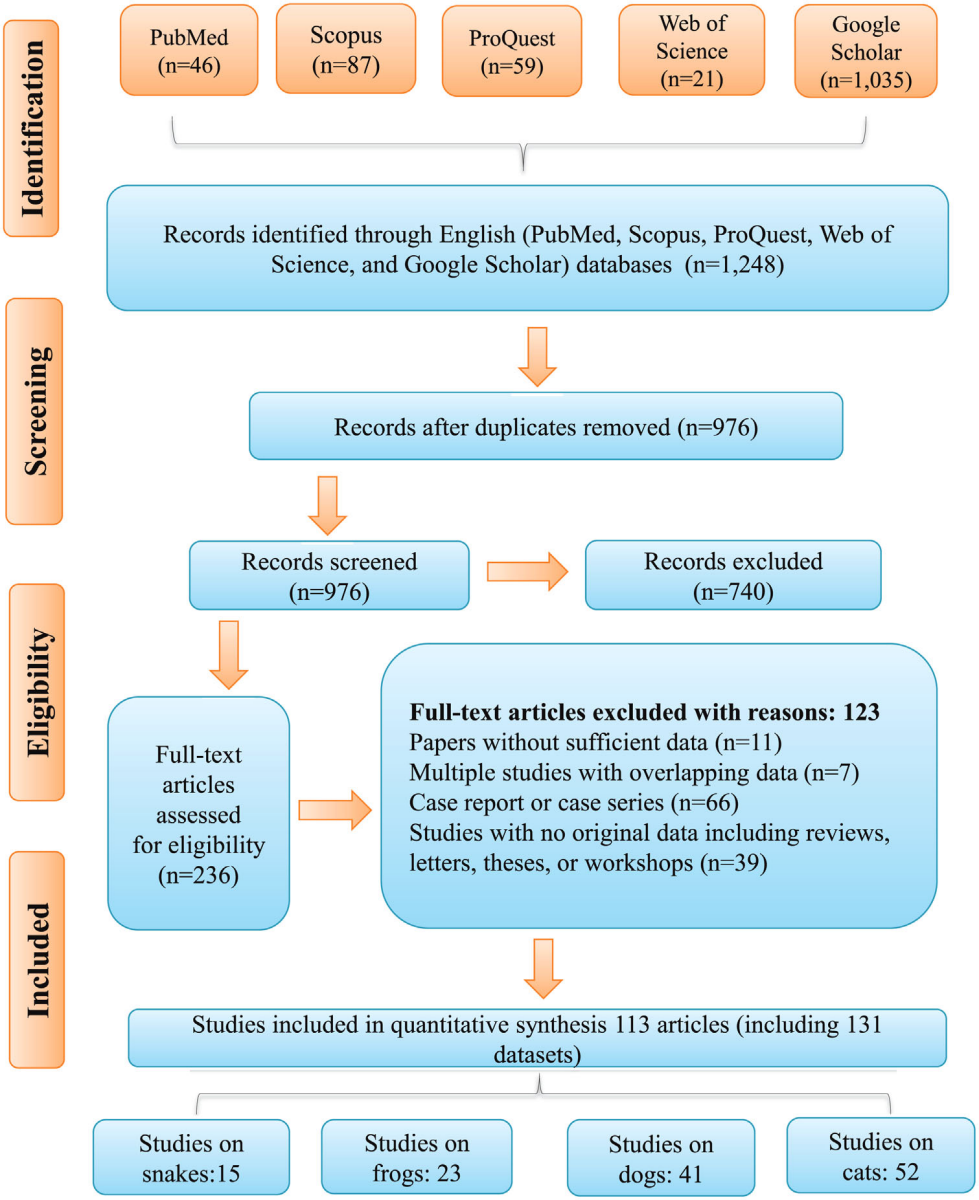
Flow diagram of the study design process

The initial database search identified 1248 articles including 46 from PubMed, 87 from Scopus, 59 from ProQuest, 21 from Web of Science and 1035 from Google Scholar, of which 59 duplicates were removed. Of 976 records screened, 740 articles excluded, as they did not meet the inclusion criteria. Of 236 full‐text articles assessed for eligibility, 125 articles were excluded with the following reasons: papers without sufficient data (*n* = 11), multiple studies with overlapping data (*n* = 7), case report or case series (*n* = 66), studies with no original data including reviews, letters, theses or workshops (*n* = 39). Finally, 113 articles (including 131 data sets) were included in the current systematic review and meta‐analysis (Figure 1).

### 3.2 Prevalence in intermediate hosts (snakes and frogs)

**FIGURE 2:**
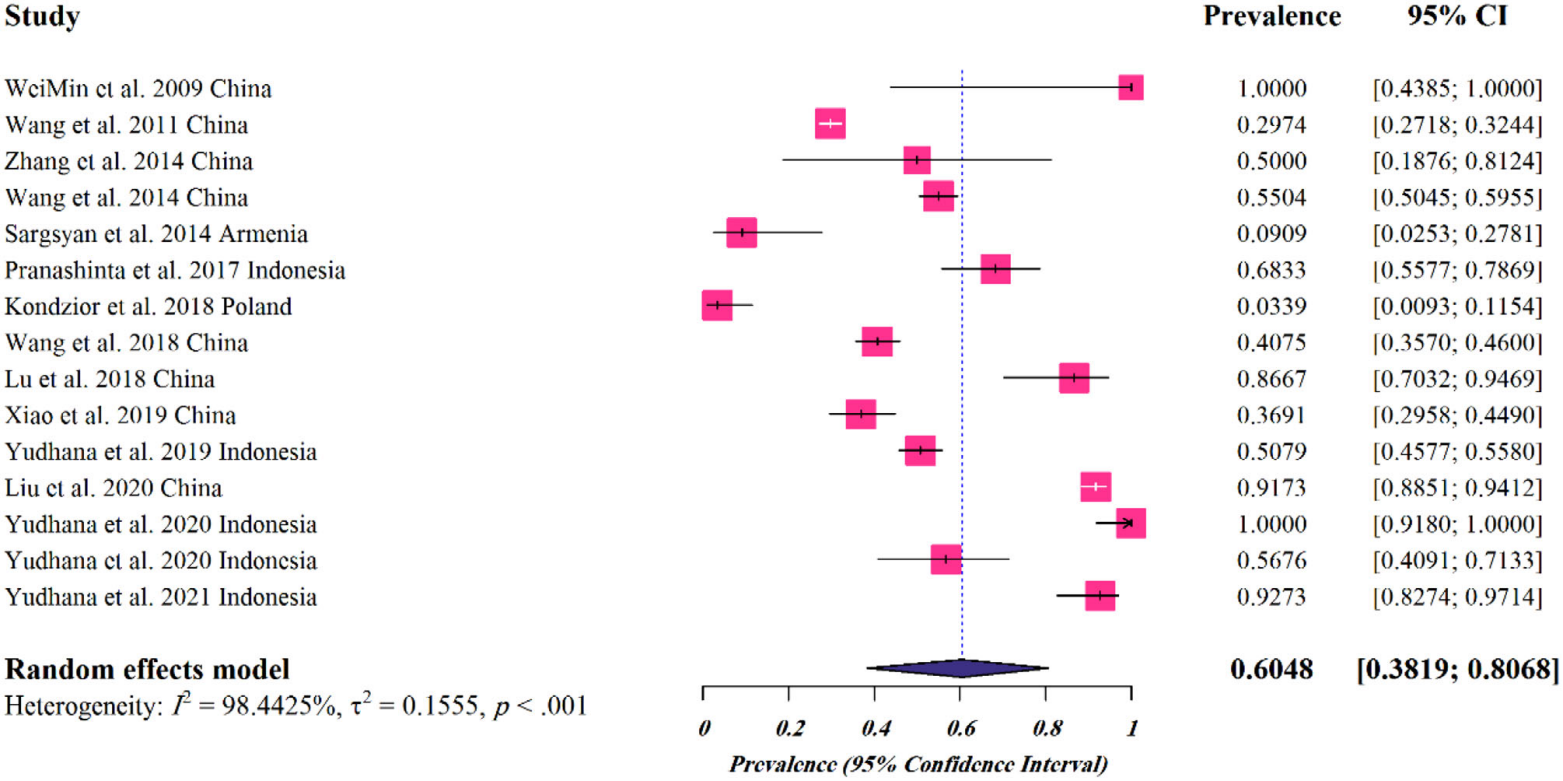
Forest plots for random‐effects meta‐analysis of *Spirometra* in snakes

For snake hosts, a total of 15 studies (3179 cases) were analysed, of which 1520 harboured *Spirometra* parasites spargana (Table 2). Global pooled prevalence rate for snakes was 0.6048% (95%CI: 0.3819–0.8068%) (Figure 2). According to the species of the parasite, the estimated pooled prevalence was as follows: 1.000% (95%CI: 0.712–1.000%) for *S. mansoni*, 0.752% (95%CI: 0.438–0.963%) for *Spirometra* spp. and 0.424% (95%CI: 0.142–0.736%) for *S. erinaceieuropaei* (Table 2). The highest prevalence was found in Asia (0.696, 95%CI: 0.502–0.859). The analyses based on different climates and climatic parameters revealed that the infection was most prevalent in regions with tropical rainforest climate (0.790, 95%CI: 0.402–0.995), average temperature of >20°C (0.790, 95%CI: 0.402–0.995), annual rainfall of >1500 mm (0.790, 95%CI: 0.402–0.995) and humidity of 40–75% (0.653, 95%CI: 0.444–0.835). The pooled prevalence rate with regard to the income level was the highest for lower‐middle income countries (0.790, 95%CI: 0.402–0.995) (Table 2).

**FIGURE 3:**
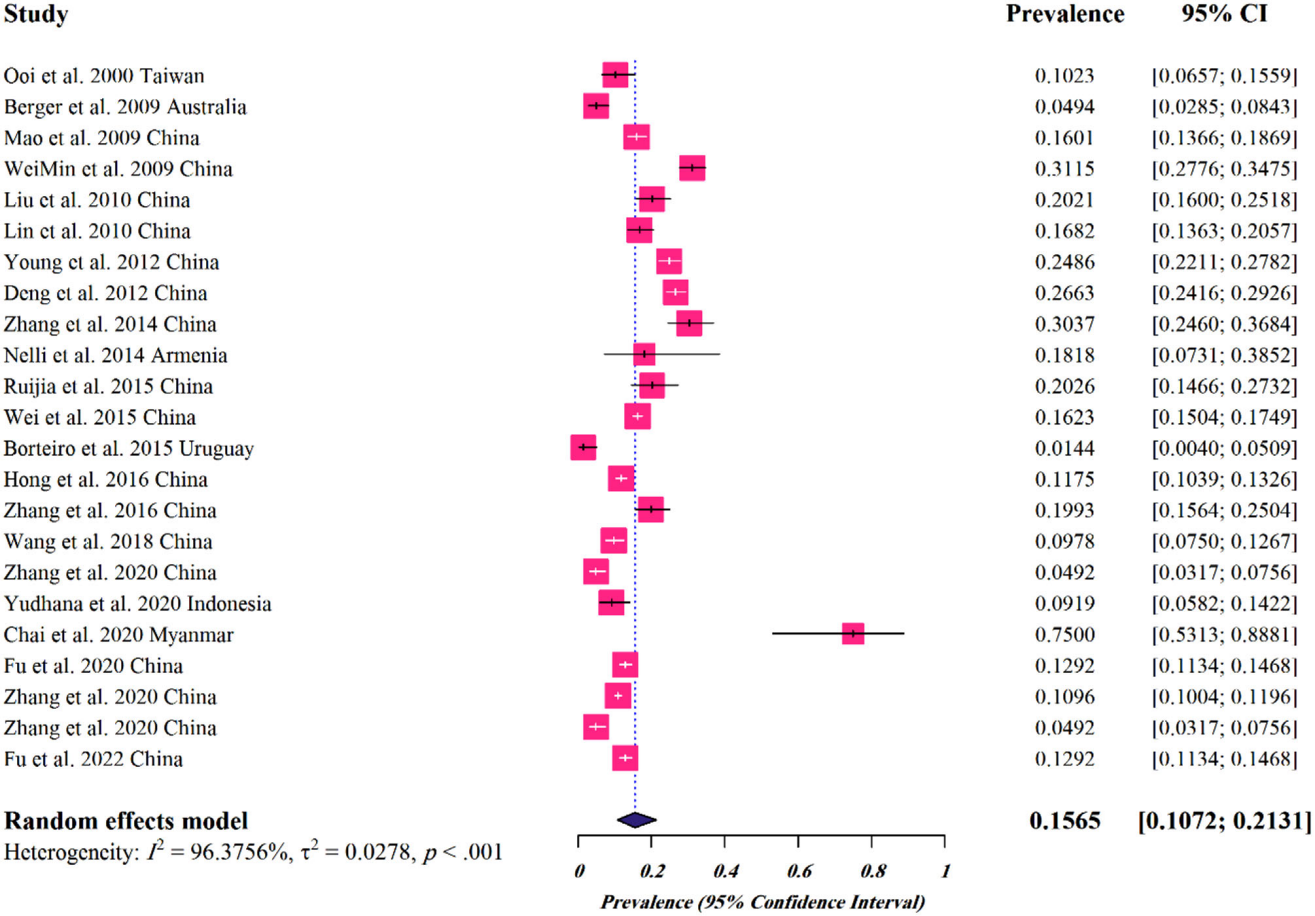
Forest plots for random‐effects meta‐analysis of *Spirometra* in frogs

For frog hosts, a total of 23 studies (19,585 cases) were analysed, of which 2948 found to be infected with *Spirometra* parasites spargana, giving a global pooled prevalence of 0.1565% (95%CI: 0.1072–0.2131%) (Table 2 and Figure 3). Regarding the species of the parasite, the estimated pooled prevalence was as follows: 0.232% (95%CI: 0–1.000%) for *Spirometra* spp., 0.202% (95%CI: 0.135–0.280%) for *S. mansoni* and 0.130% (95%CI: 0.089–0.177) for *S. erinaceieuropaei* (Table 2). The highest prevalence rate was related to Asia (0.181, 95%CI: 0.052–0.365). The analyses based on different climates and climatic parameters revealed that the infection was most prevalent in regions with Tropical monsoon climate (0.750, 95%CI: 0.542–0.910), average temperature of >20°C (0.273, 95%CI: 0–1.000), annual rainfall of <400 mm (0.181, 95%CI: 0.052–0.365) and humidity of 40–75% (0.156, 95%CI: 0.107–0.213). The pooled prevalence rate with regard to the income level was highest for lower‐middle income countries (0.385, 95%CI: 0–1.000) (Table 2).

### 3.3 Prevalence in definitive hosts (dogs and cats)

**FIGURE 4:**
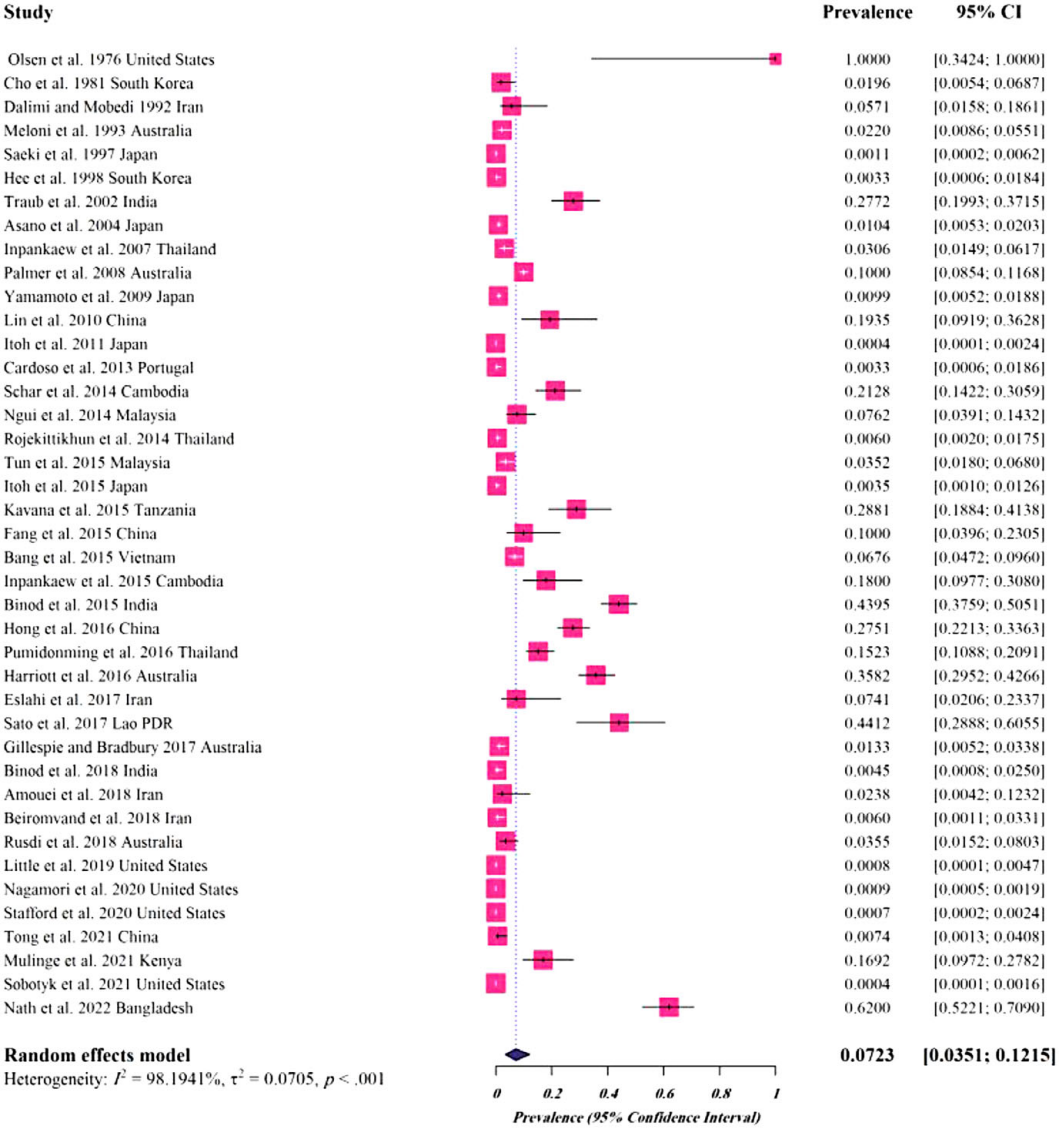
Forest plots for random‐effects meta‐analysis of *Spirometra* in dogs

For dog hosts, a total of 41 studies (28,101 cases) were analysed, of which 689 harboured *Spirometra* parasites, giving a pooled prevalence of 0.0723% (95%CI: 0.0351–0.1215%) (Table 2 and Figure 4). Regarding the species of the parasite, the estimated pooled prevalence was as follows: 0.141% (95%CI: 0–0.519%) for *S. mansoni*, 0.080% (95%CI: 0.029–0.154%) for *Spirometra* spp. and 0.045% (95%CI: 0.006–0.116%) for *S. erinaceieuropaei* (Table 2). The highest prevalence rate was related to Africa (0.224%, 95%CI: 0–0.971%). The analyses based on different climates and climatic parameters revealed that the infection was most prevalent in regions with Tropical monsoon climate (0.250%, 95%CI: 0.034–0.577%), average temperature of >20°C (0.162%, 95%CI: 0.074–0.275%), annual rainfall of >1500 mm (0.157%, 95%CI: 0–0.650%) and humidity of 40–75% (0.078%, 95%CI: 0.031–0.143%). The pooled prevalence rate with regard to the income level was highest for low‐income countries (0.288%, 95%CI: 0.180–0.409%) (Table 2).

**FIGURE 5:**
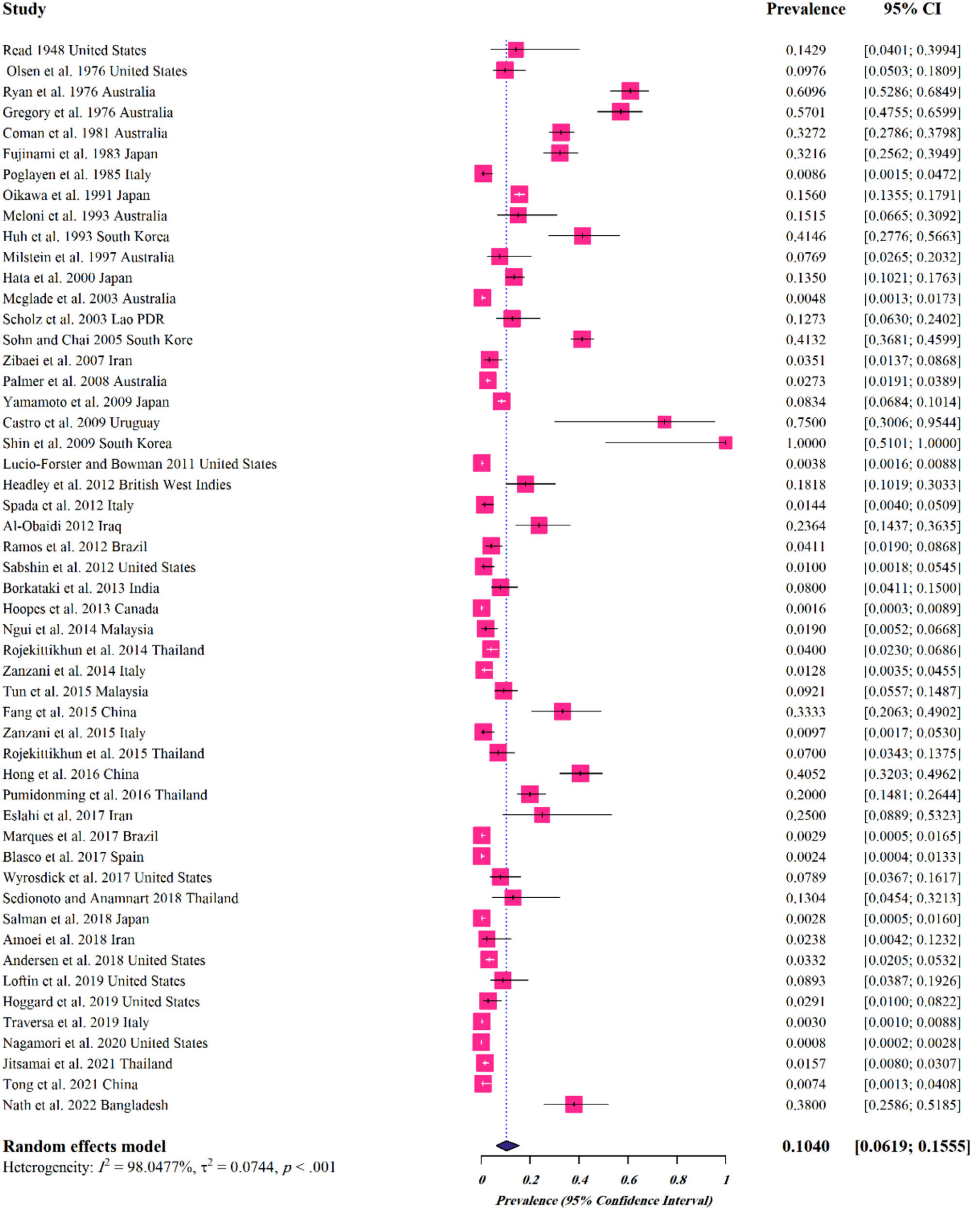
Forest plots for random‐effects meta‐analysis of *Spirometra* in cats

For cat hosts, a total of 52 studies (15,631 cases) were analysed, of which 1131 were infected with *Spirometra* parasites, giving a pooled prevalence of 0.1040% (95%CI: 0.0619–0.1555%) (Table 2 and Figure 5). Based on the species of the parasite, the estimated pooled prevalence was as follows: 0.268% (95%CI: 0.123–0.445%) for *S. erinaceieuropaei*, 0.094% (95%CI: 0–0.314%) for *S. mansoni*, 0.051% (95%CI: 0.001–0.164%) for *S. mansonoides*, 0.049% (95%CI: 0.020–0.089%) for *Spirometra* spp. (Table 2). The highest prevalence rate was related to Oceania (0.203%, 95%CI: 0.026–0.487%). The analyses with regard to different climates and climatic parameters revealed that the infection was most prevalent in regions with Oceanic climate (0.203%, 95%CI: 0.026–0.487%), average temperature of 10–20°C (0.115%, 95%CI: 0.057–0.190%), annual rainfall of >1500 mm (0.128%, 95%CI: 0–0.733%) and humidity of 40–75% (0.113%, 95%CI: 0.063–0.175%). The pooled prevalence rate with regard to the income level was highest for lower‐middle income countries (0.134%, 95%CI: 0.039–0.273%) (Table 2).

### 3.4 Prevalence based on diagnostic methods

There were two diagnostic procedures used for the detection of adult and spargana of *Spirometra* spp. in included studies (Table 2). All studies on intermediate hosts (snakes and frogs) were conducted on carcasses, while studies on definitive hosts (dogs and cats) were performed on stool specimens or carcasses. Totally 112 studies used morphological detection method and 19 studies used molecular detection method. According to the diagnostic method, the highest pooled prevalence was related to morphological method for studies on snakes, frog and cats with rate of 0.665% (95%CI: 0.349–0.915%), 0.189% (95%CI: 0.105–0.290%) and 0.104% (95%CI: 0.061–0.155%), respectively. However, all studies on cats were performed via morphological method (Table 2). Regarding studies on dogs, the highest pooled prevalence was observed for molecular technique (0.101%, 95%CI: 0–0.854%) (Table 2).

### 3.5 Meta regression analysis

Heterogeneity was noted for the year of publication and average temperature. Accordingly, the results of the test were significant for the year of publication for studies on cats (slop = 0.0069, *p* < 0.0062), and average temperature for studies on dogs (slop = 0.0149, *p* < 0.0172) (Supplementary Figure S1).

### 3.6 Publication bias

Asymmetry of the funnel plot indicates that publication bias was present in studies on cats (Egger's test: *t* = 3.31, *p* = 0.0017, and Begg's test: *p* = 0.0043) and dogs (Egger's test: *t* = 5.30, *p* = 0.0001, and Begg's test: *p* = 0.0003). There was no statistical publication bias for studies in snakes and frogs (Supplementary Figure S2A1–B4). Furthermore, asymmetrical Doi plots suggest presence of publication bias for prevalence in snakes, dogs and cats. Accordingly, there was major asymmetry for snakes (LFK index  = 2.93), dogs (LFK index  = 5.32) and cats (LFK index  =  3.57). In contrast, there was no asymmetry for prevalence in frogs (LFK index =  0.92) (Supplementary Figure S2C1–C4).

**FIGURE 6:**
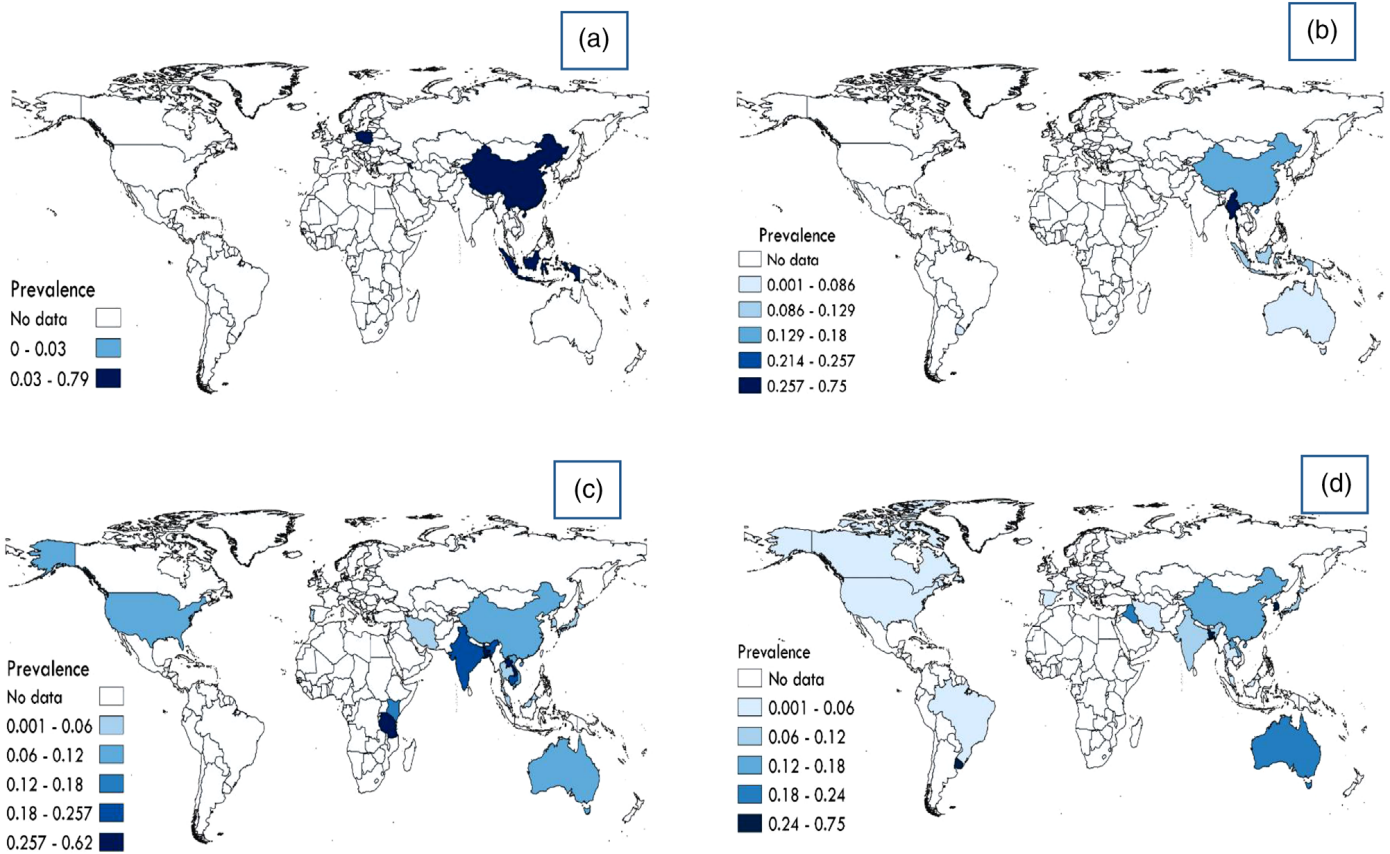
Prevalence of *Spirometra* in (a) snakes, (b) frogs, (c) dogs and (d) cats in different geographical regions of the world based on the included studies

A QGIS3 software (version 3.1) was used to provide a map representing the global prevalence of *Spirometra* in snakes, frogs, dogs and cats in different geographical regions of the world based on included studies (Figure 6)

## 4 DISCUSSION

The current systematic review and meta‐analysis aims to estimate the global prevalence of *Spirometra* infection in snakes, frogs, dogs and cats. The overall prevalence of *Spirometra* in intermediate hosts and definitive hosts was found to be 0.313% and 0.089%, respectively. Among intermediate hosts analysed in this study, *Spirometra* infection was more prevalent in Asia with higher rate in snakes (0.6048%) than in frogs. Occurrence of infection with plerocercoid in snakes is more probably caused by ingestion of infected frogs than acquisition of the procercoid (Oda et al., 2016). In reptile hosts subcutaneous tissues and muscles are the frequent sites involved with infective larvae of the parasite (Mendoza‐Roldan et al., 2020). Snakes are the most common reptiles to be an intermediate host for *Spirometra*, a best‐known reptile‐borne zoonotic tapeworm. The raw or undercooked snakes meat for consumption or for purposes such as zootherapeutic remedies are regarded as a rout for transmission of sparganosis (Magnino et al., 2009; Mendoza‐Roldan et al., 2020).

One of the causes for the growth in reports of foodborne diseases in recent years has been the rising demand for animal protein, as well as, exotic and raw foods resulted in the expansion of some farming systems (wildlife farming) in emerging or underdeveloped nations where health monitoring may be inadequately managed (Broglia & Kapel, 2011; Xiao et al., 2021).

The complex life cycle of *Spirometra* parasites gives them the opportunity to be acquired not only via consumption of raw wild animal products (e.g. snakes and frogs infected with plerocercoids), but also by drinking contaminated water containing infected copepods (water‐borne route) (Yudhana et al., 2021).

It has been documented that the consumption of wild meat (bushmeat) has a direct relationship with poverty and low‐income communities, where is a lack of sanitary drinking water sources, sanitation and hygiene (Badri et al., 2022; Eslahi et al., 2021; Kouassi et al., 2019; Maleki et al., 2020; Prüss‐Ustün et al., 2014; Van Velden et al., 2020). Also, water hygienic interventions in low‐ and low‐middle‐income settings, which place less emphasis on limiting animal faeces exposure in water sources, help to maintain parasitic remains and provide the water‐borne cycle (Delahoy et al., 2018).

In Southeast Asian countries over the last decades, there has been a growing interest in snakes, and despite the negative impact on wildlife, snake farming and the international trade of snakes have emerged as significant phenomena. It is mostly visible in the Chinese population relative to the involvement of snakes in their dietary habits (Aust et al., 2017; Xiao et al., 2019).

Furthermore, sushi and sashimi as popular dishes prepared from the meat of frogs and snakes are the major sources of human sparganosis in Southeast Asia, where there is still a lack of awareness relating to the risk of this infection (Nawa et al., 2005).

Among the definitive hosts we analysed in the current study, cats have a higher infection rate (0.1040%) than dogs. This finding suggests that cats are the potential sources of maintenance for tapeworms of the genus *Spirometra*. They have a significant role in environmental contamination, transmission of many microbial pathogens and can serve as reservoirs for several parasites of both public and wildlife health importance (Hernandez et al., 2018; Jeon et al., 2018; Taghipour et al., 2021). Cats are predators of a broad variety of prey, including amphibians and reptiles. Thus, it is believed that the prevalence of *Spirometra* parasites is impacted by host diets and the access of definitive hosts to infected intermediate hosts (Hernandez et al., 2018).

The identification of both larval stage and adults of *Spirometra* species is through morphological and molecular approaches (Jeon et al., 2018). The morphological identification of *Spirometra* tapeworms to the species level is based on taxonomic differences (Badri et al., 2017). Recent advances of molecular techniques promote species identification for both adults and larvae. These techniques that rely on DNA sequencing of the whole mitochondrial COI (cytochrome c oxidase subunit I) gene are considered as the only approach to precisely specify the species (Kuchta et al., 2021). However, it is dependent on the availability of gene sequences and the accuracy of data, especially in the case that parasites are lacerated in the host's cadaver due to the road‐killing incidences (Eslahi et al., 2021; Tang et al., 2017).

Asia was the most prevalent region for *Spirometra* infection in snakes and frogs, whereas Africa and Oceania had been shown to have the highest pooled prevalence rates in dogs and cats, respectively. The geographical variation found for the prevalence emphasises that the infection risk is different in each region. *Spirometra* tapeworms have a broad host spectrum and they have been reported in domesticated and wild animals from different geographical regions all over the world (except Antarctica) (Bagrade et al., 2021). Although, this infection is mostly observed in tropical and sub‐tropical areas with the highest prevalence in South‐east Asia and East Africa (Farrar et al., 2013; L. N. Liu et al., 2015). This statement is in consistent with our analyses suggesting that oceanic and tropical climates present the highest prevalence for the infection.

The persistence of the heteroxenous life cycle and survival of *Spirometra* are affected by environmental factors including physiochemical conditions (pH, pCO_2_, O_2_, viscosity) and temperature (Muller & Wakelin, 2002). Moreover, humid areas with abundant river networks offer an optimal condition for development of *Spirometra* parasites (Marta Kołodziej‐Sobocińska et al., 2019). However, plerocercoids are able to tolerate stress conditions, such as diverse range of pH. As well, they have ability to survive in various vertebrate hosts even cold‐blooded ones, except fish (Kavana, 2015; Muller & Wakelin, 2002).

Given that the information on distinct morphological traits of both adults and plerocercoids are limited and there were lack of molecular diagnostics at the time of the study, most reports cannot identify *Spirometra* to the species level. Regardless of the fact that several species of the parasite have been identified, the taxonomy of *Spirometra* tapeworms is still unclear and needs to be more clarified (Bagrade et al., 2021).

The results of the present systematic review and meta‐analysis should be interpreted cautiously referring to some of the limitations. First, our analyses may have been affected by publication bias, as the result of absence or the low number of published studies from some geographic regions. Second, there were several single case reports of *Spirometra* species other than those included in the current study. Another point is the fact that this study was limited to publications in English. Finally, there were small‐study effects in some studies we included in our analyses attributable to (1) small sample size, (2) sampling bias relating to the nature of sample collection from wildlife over several years and (3) lack of a sensitive diagnostic technique. Despite these limitations, this study provides the most comprehensive estimates of the prevalence of *Spirometra* infection in snakes, frogs, dogs and cats from a global perspective.

## 5 CONCLUSION

The findings of the current systematic review and meta‐analysis indicate the relatively significant burden and current status of *Spirometra* infection in snakes, frogs, dogs and cats in different parts of the world and highlight the importance of conducting investigations in more geographical regions. Furthermore, our results showed that this infection may represent a significant risk for public health, especially in low‐ and lower‐middle‐income countries and in regions with oceanic and tropical climates. Paying attention to preventive strategies such as protection of aquaculture systems from being contaminated with faeces of dogs and cats, development of precise diagnostic approaches for foodborne parasitic infection during preparation, distribution, and selling stages, improving public education regarding the hazard of consuming reptile and amphibian products. Moreover, breaking the life cycle of the parasites and decreasing the burden of infective larvae in the aquatic environment play a key role in a large‐scale control measure in endemic regions.

## AUTHOR CONTRIBUTIONS

MB, LMMDC, MFH, and AVE contributed to study design. AK, LZ, FB and PM searched for primary publications, screened and appraised primary studies. MB and AVE extracted the data and wrote the study manuscript. MO contributed to data analysis. All authors read the manuscript and participated in the preparation of the final version of the manuscript.

## CONFLICT OF INTEREST

The authors declare that there are no conflicts of interests.

## ETHICAL APPROVAL

None required.

### PEER REVIEW

The peer review history for this article is available at https://publons.com/publon/10.1002/vms3.932.

## Supporting information

Supporting Information

Supporting Information

Supporting Information

## Data Availability

The authors confirm that the data supporting the findings of this study are available within the article and its supplementary material.
