# Supplementary material for: The global prevalence of Spirometra parasites in snakes, frogs, dogs, and cats: A systematic review and meta‐analysis
Source: Vet Med Sci. 2022 Sep 9;8(6):2785–805. doi: 10.1002/vms3.932 (PMC9677416; doi:10.1002/vms3.932)
Supplement: Supplementary file 1 — Supporting Information [file VMS3-8-2785-s001.pdf]

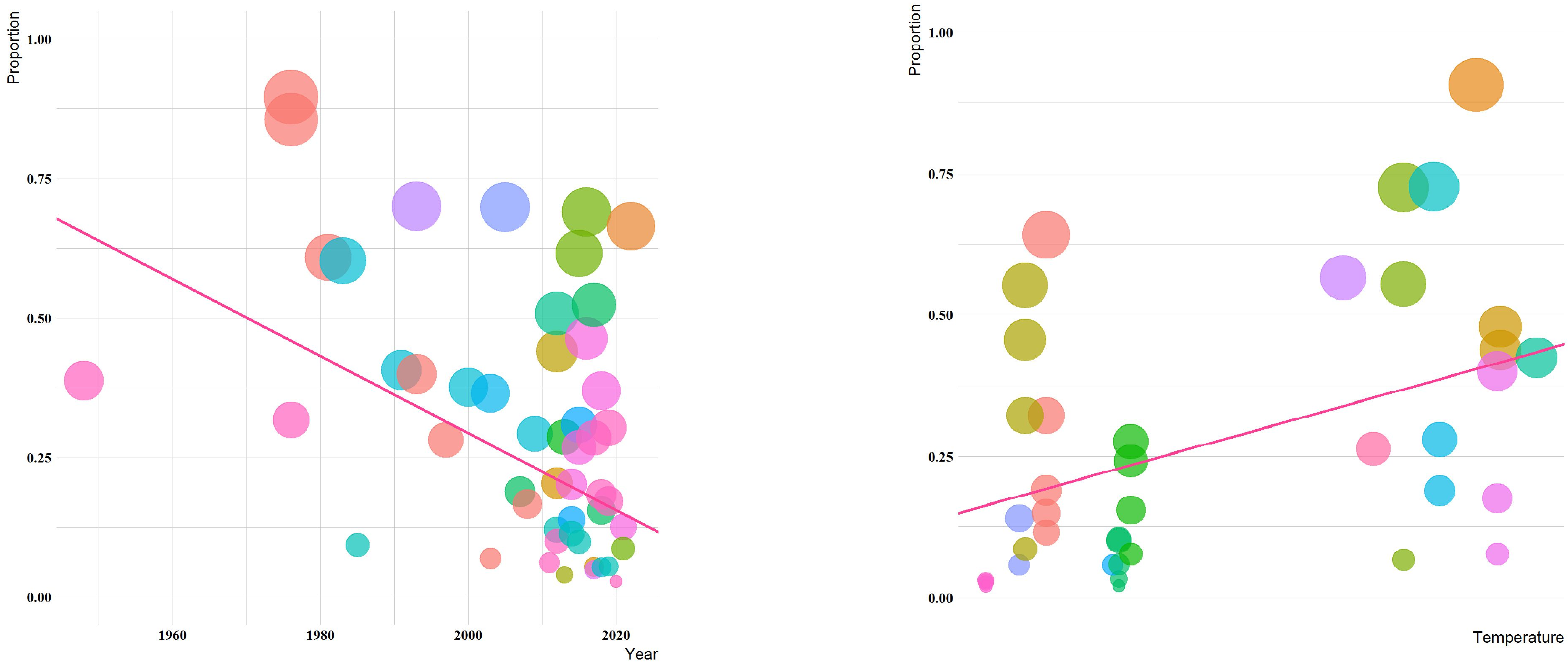

Supplementary Figure 1. A meta-regression graph for *Spirometra* in cats (year of publication), and dogs (average temperature). (The pink line is the regression line, which was plotted based on the intercept and the slope of the regression model. The different color bubbles represent the countries under study and their sizes indicate the effect size of each study).
