# Supplementary material for: The global prevalence of Spirometra parasites in snakes, frogs, dogs, and cats: A systematic review and meta‐analysis
Source: Vet Med Sci. 2022 Sep 9;8(6):2785–805. doi: 10.1002/vms3.932 (PMC9677416; doi:10.1002/vms3.932)
Supplement: Supplementary file 2 — Supporting Information [file VMS3-8-2785-s002.pdf]

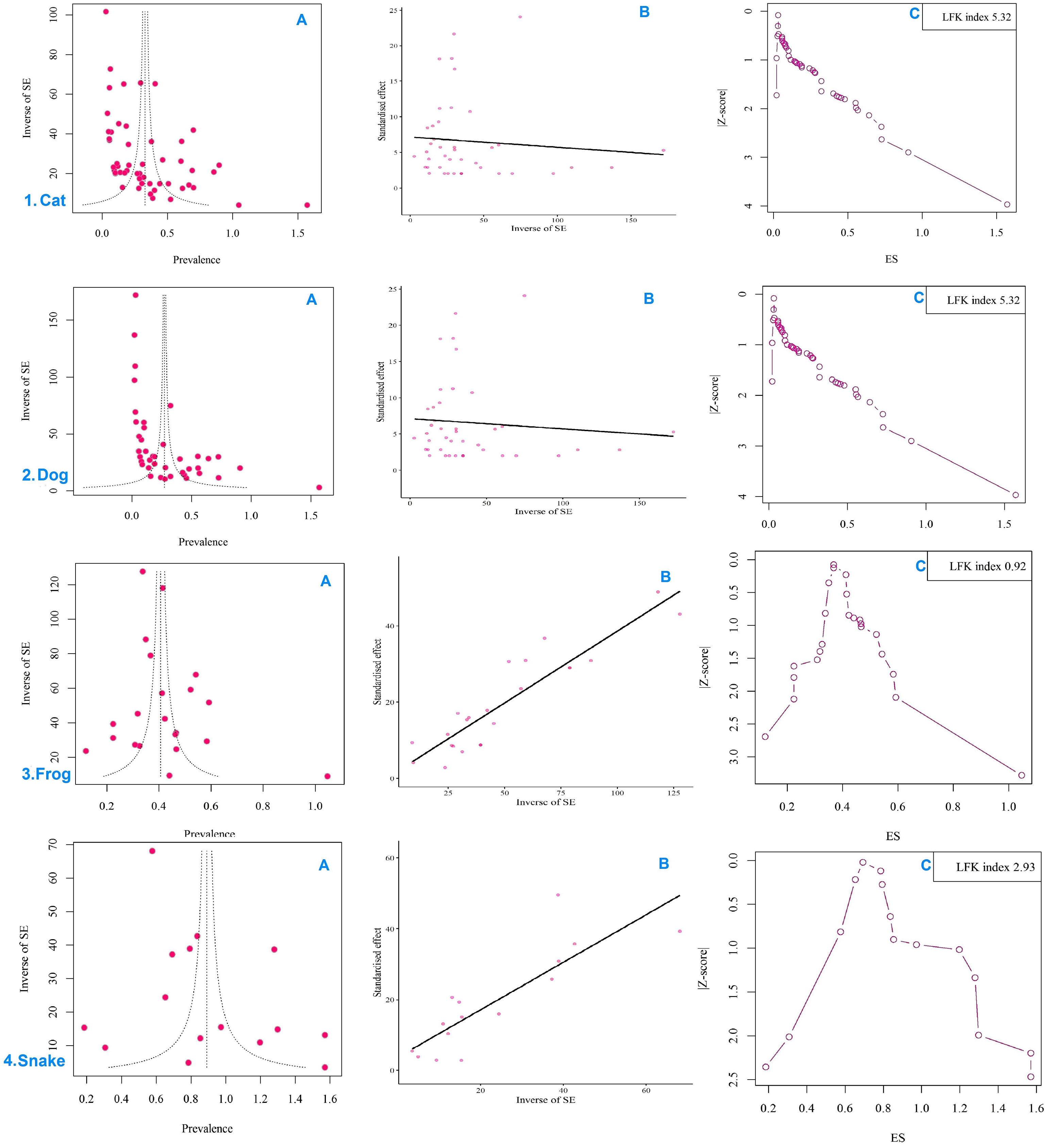

Supplementary Figure 2. Egger's funnel plot (A), and Begg's funnel plot (B) to assess publication bias in studies evaluating *Spirometra* in cat (1), dogs (2), frogs (3), and snakes (4) (Colored circles represent each study. The middle line is the effect size and the other two lines are the corresponding confdence ranges). Doi plot (C) for the global *Spirometra* in cats, dogs, frogs, and snakes.
