# Supplementary material for: The global prevalence of Spirometra parasites in snakes, frogs, dogs, and cats: A systematic review and meta‐analysis
Source: Vet Med Sci. 2022 Sep 9;8(6):2785–805. doi: 10.1002/vms3.932 (PMC9677416; doi:10.1002/vms3.932)
Supplement: Supplementary file 3 — Supporting Information [file VMS3-8-2785-s003.docx]

**Supplementary Table 1.** Quality assessment using the Newcastle–Ottawa scale modified for cross-sectional studies

| No. | First author | Year | Selection  (maximum of 5 stars) | Comparability  (maximum of 2 stars) | Outcome  (maximum of 3 stars) | Total Score |
| --- | --- | --- | --- | --- | --- | --- |
| 1 | Read | 1948 | ******* | ***** | ******* | 7 |
| 2 | Olsen et al. | 1976 | ******* | ***** | ****** | 6 |
| 3 | Ryan et al. | 1976 | ******* | ****** | ****** | 7 |
| 4 | Gregory et al. | 1976 | ****** | ****** | ******* | 7 |
| 5 | Coman et al. | 1981 | ******* | ***** | ******* | 7 |
| 6 | Fujinami et al. | 1983 | ******* | ***** | ******* | 7 |
| 7 | Poglayen et al. | 1985 | ******* | ****** | ******* | 8 |
| 8 | Oikawa et al. | 1991 | ******* | ***** | ******* | 7 |
| 9 | Meloni et al. | 1993 | ******* | ****** | ****** | 7 |
| 10 | Huh et al. | 1993 | ******* | ***** | ******* | 7 |
| 11 | Milstein et al. | 1997 | ******** | ***** | ******* | 8 |
| 12 | Hata et al. | 2000 | ******* | ***** | ******* | 7 |
| 13 | Mcglade et al. | 2003 | ****** | ***** | ****** | 5 |
| 14 | Scholz et al. | 2003 | ****** | ****** | ****** | 5 |
| 15 | Sohn and Chai | 2005 | ******** | ****** | ****** | 8 |
| 16 | Zibaei et al. | 2007 | ******* | ****** | ****** | 7 |
| 17 | Palmer et al. | 2008 | ******* | ****** | ******* | 8 |
| 18 | Yamamoto et al. | 2009 | ******* | ****** | ******* | 8 |
| 19 | Castro et al. | 2009 | ******** | ****** | ****** | 8 |
| 20 | Shin et al. | 2009 | ****** | ****** | ****** | 6 |
| 21 | Lucio-Forster and Bowman | 2011 | ****** | ****** | ****** | 6 |
| 22 | Headley et al. | 2012 | ******* | ***** | ****** | 6 |
| 23 | Spada et al. | 2012 | ******* | ***** | ****** | 6 |
| 24 | Al-Obaidi | 2012 | ******** | ****** | ******* | 9 |
| 25 | Sabshin et al. | 2012 | ******* | ****** | ****** | 7 |
| 26 | Borkataki et al. | 2013 | ******* | ****** | ******* | 8 |
| 27 | Ramos et al. | 2013 | ******* | ****** | ****** | 7 |
| 28 | Hoopes et al. | 2013 | ******* | ****** | ******* | 8 |
| 29 | Ngui et al. | 2014 | ****** | ****** | ****** | 6 |
| 30 | Rojekittikhun et al. | 2014 | ******** | ***** | ******* | 8 |
| 31 | Zanzani et al. | 2014 | ******** | ****** | ****** | 8 |
| 32 | Tun et al. | 2015 | ******** | ***** | ****** | 7 |
| 33 | Fang et al. | 2015 | ****** | ****** | ******* | 7 |
| 34 | Zanzani et al. | 2015 | ****** | ****** | ****** | 6 |
| 35 | Rojekittikhun et al. | 2015 | ****** | ****** | ****** | 5 |
| 36 | Hong et al. | 2016 | ******** | ****** | ******* | 9 |
| 37 | Pumidonming et al. | 2016 | ******* | ***** | ****** | 6 |
| 38 | Eslahi et al. | 2017 | ****** | ****** | ******* | 7 |
| 39 | Marques et al. | 2017 | ******* | ****** | ****** | 7 |
| 40 | Blasco et al. | 2017 | ******* | ****** | ****** | 7 |
| 41 | Wyrosdick et al. | 2017 | ******** | ***** | ****** | 7 |
| 42 | Sedionoto and Anamnart | 2018 | ******* | ****** | ****** | 7 |
| 43 | Salman et al. | 2018 | ******** | ***** | ***** | 6 |
| 44 | Amoei et al. | 2018 | ******** | ***** | ****** | 7 |
| 45 | Andersen et al. | 2018 | ****** | ****** | ****** | 5 |
| 46 | Loftin et al. | 2019 | ******* | ****** | ******* | 8 |
| 47 | Hoggard et al. | 2019 | ******* | ****** | ****** | 7 |
| 48 | Traversa et al. | 2019 | ****** | ****** | ****** | 6 |
| 49 | Nagamori et al. | 2020 | ******* | ****** | ***** | 6 |
| 50 | Jitsamai et al. | 2021 | ******* | ****** | ****** | 7 |
| 51 | Tong et al. | 2021 | ****** | ****** | ****** | 6 |
| 52 | Nath et al. | 2022 | ****** | ****** | ******* | 7 |
| 53 | Cho et al. | 1981 | ******** | ****** | ****** | 8 |
| 54 | Dalimi and Mobedi | 1992 | ******* | ***** | ******* | 7 |
| 55 | Saeki et al. | 1997 | ******** | ***** | ******* | 8 |
| 56 | Hee et al. | 1998 | ****** | ****** | ****** | 6 |
| 57 | Traub et al. | 2002 | ******* | ****** | ****** | 7 |
| 58 | Asano et al. | 2004 | ******* | ***** | ******* | 7 |
| 59 | Inpankaew et al. | 2007 | ******* | ****** | ****** | 7 |
| 60 | Lin et al. | 2010 | ****** | ****** | ******* | 7 |
| 61 | Itoh et al. | 2011 | ******** | ****** | ******* | 9 |
| 62 | Cardoso et al. | 2013 | ****** | ****** | ****** | 6 |
| 63 | Schar et al. | 2014 | ******* | ***** | ******* | 7 |
| 64 | Itoh et al. | 2015 | ******** | ***** | ******* | 8 |
| 65 | Kavana et al. | 2015 | ******* | ****** | ****** | 7 |
| 66 | Bang et al. | 2015 | ******* | ***** | ******* | 7 |
| 67 | Inpankaew et al. | 2015 | ******* | ****** | ****** | 7 |
| 68 | Binod et al. | 2015 | ****** | ****** | ******* | 7 |
| 69 | Harriott | 2016 | ******** | ***** | ******* | 8 |
| 70 | Sato et al. | 2017 | ****** | ***** | ******* | 6 |
| 71 | Gillespie and Bradbury | 2017 | ******* | ***** | ******* | 7 |
| 72 | Binod et al. | 2018 | ******* | ***** | ****** | 6 |
| 73 | Beiromvand et al. | 2018 | ******** | ****** | ******* | 9 |
| 74 | Rusdi et al. | 2018 | ****** | ****** | ****** | 6 |
| 75 | Little et al. | 2019 | ******* | ***** | ******* | 7 |
| 76 | Stafford et al. | 2020 | ******** | ***** | ******* | 8 |
| 77 | Mulinge et al. | 2021 | ******* | ****** | ****** | 7 |
| 78 | Sobotyk et al. | 2021 | ******* | ***** | ******* | 7 |
| 79 | Ooi et al. | 2000 | ******* | ****** | ****** | 7 |
| 80 | Berger et al. | 2009 | ****** | ****** | ******* | 7 |
| 81 | Mao et al. | 2009 | ******** | ***** | ******* | 8 |
| 82 | WeiMin et al. | 2009 | ****** | ***** | ******* | 6 |
| 83 | Liu et al. | 2010 | ******* | ***** | ******* | 7 |
| 84 | Lin et al. | 2010 | ******* | ***** | ****** | 6 |
| 85 | Young et al. | 2012 | ******* | ****** | ******* | 8 |
| 86 | Deng et al. | 2012 | ******** | ****** | ******* | 9 |
| 87 | Zhang et al. | 2014 | ****** | ****** | ****** | 6 |
| 88 | Nelli et al. | 2014 | ****** | ***** | ******* | 6 |
| 89 | Ruijia et al. | 2015 | ******* | ***** | ******* | 7 |
| 90 | Wei et al. | 2015 | ******* | ***** | ****** | 6 |
| 91 | Borteiro et al. | 2015 | ******** | ****** | ******* | 9 |
| 92 | Hong et al. | 2016 | ****** | ****** | ****** | 6 |
| 93 | Zhang et al. | 2016 | ******* | ***** | ******* | 7 |
| 94 | Wang et al. | 2018 | ******** | ***** | ******* | 8 |
| 95 | Zhang et al. | 2020 | ******* | ****** | ****** | 7 |
| 96 | Yudhana et al. | 2020 | ******* | ***** | ******* | 7 |
| 97 | Chai et al. | 2020 | ******* | ****** | ****** | 7 |
| 98 | Fu et al. | 2020 | ****** | ****** | ******* | 7 |
| 99 | Zhang et al. | 2020 | ******** | ***** | ******* | 8 |
| 100 | Zhang et al. | 2020 | ****** | ***** | ******* | 6 |
| 101 | WeiMin et al. | 2009 | ****** | ***** | ******* | 6 |
| 102 | Wang et al. | 2011 | ******* | ***** | ******* | 7 |
| 103 | Wang et al. | 2014 | ******* | ***** | ******* | 7 |
| 104 | Sargsyan et al. | 2014 | ******* | ***** | ****** | 6 |
| 105 | Pranashinta et al. | 2017 | ******** | ****** | ******* | 9 |
| 106 | Kondzior et al. | 2018 | ****** | ****** | ****** | 6 |
| 107 | Lu et al. | 2018 | ****** | ***** | ******* | 6 |
| 108 | Xiao et al. | 2019 | ******* | ***** | ******* | 7 |
| 109 | Yudhana et al. | 2019 | ******* | ***** | ****** | 6 |
| 110 | Liu et al. | 2020 | ******** | ****** | ******* | 9 |
| 111 | Yudhana et al. | 2020 | ****** | ****** | ****** | 6 |
| 112 | Yudhana et al. | 2021 | ******** | ***** | ******* | 8 |
| 113 | Fu et al. | 2022 | ****** | ****** | ****** | 6 |

*Indicates one criteria was followed, ** two criteria were followed, ***three criteria were followed, ****four criteria were followed, and ***** five criteria were followed
